# Immune durability and protection against SARS-CoV-2 re-infection in Syrian hamsters

**DOI:** 10.1080/22221751.2022.2058419

**Published:** 2022-04-18

**Authors:** C. J. Field, T. A. Heinly, D. R. Patel, D. G. Sim, E. Luley, S. L. Gupta, T. H. Vanderford, J. Wrammert, T. C. Sutton

**Affiliations:** a Department of Veterinary and Biomedical Science, The Pennsylvania State University, University Park, PA, USA; b The Huck Institutes of Life Sciences, The Pennsylvania State University, University Park, PA, USA; c Emory-UGA Center of Excellence of Influenza Research and Surveillance (CEIRS), University Park, PA, USA; d Department of Biology, The Pennsylvania State University, University Park, PA, USA; e Animal Diagnostic Lab, The Pennsylvania State University, University Park, PA, USA; f Department of Pediatrics, Division of Infectious Disease, School of Medicine, Emory University, Atlanta, GA, USA; g Division of Microbiology and Immunology, Yerkes National Primate Research Center, Emory University, Atlanta, GA, USA

**Keywords:** SARS-CoV-2, antibodies, re-infection, hamster, immunity

## Abstract

Severe acute respiratory syndrome coronavirus-2 (SARS-CoV-2) has caused a pandemic. As immunity to endemic human coronaviruses (i.e. NL63 or OC43) wanes leading to re-infection, it was unknown if SARS-CoV-2 immunity would also decline permitting repeat infections. Recent case reports confirm previously infected individuals can become re-infected; however, re-infection may be due to heterogeneity in the initial infection or the host immune response, or may be the result of infection with a variant strain that escapes pre-existing immunity. To control these variables, we utilized the Syrian hamster model to evaluate the duration of immunity and susceptibility to re-infection with SARS-CoV-2. Hamsters were given a primary mock or SARS-CoV-2 infection (culture media or 10^5^ TCID50 USA/WA1/2020 isolate, respectively). Mock and SARS-CoV-2 infected hamsters were then given a secondary SARS-CoV-2 infection at 1, 2, 4, or 6 months post-primary infection (*n* = 14/time point/group). After the primary SARS-CoV-2 infection, hamsters developed anti-spike protein IgG, IgA, and neutralizing antibodies, and these antibodies were maintained for at least 6 months. Upon secondary SARS-CoV-2 challenge, previously SARS-CoV-2 infected animals were protected from weight loss, while all previously mock-infected animals became infected and lost weight. Importantly, despite having high titres of antibodies, one SARS-CoV-2 infected animal re-challenged at 4 months had a breakthrough infection with replicating virus in the upper and lower respiratory tract. These studies demonstrate immunity to SARS-CoV-2 is maintained for 6 months; however, protection may be incomplete and, even in the presence of high antibody titres, previously infected hosts may become re-infected.

## Introduction

Severe acute respiratory syndrome coronavirus 2 (SARS-CoV-2), the causative agent of coronavirus disease 2019 (COVID19), has caused a global pandemic [1]. Symptoms of COVID19 include fever, cough, and shortness of breath, which may lead to pneumonia and mortality in severe cases [2]. Longitudinal cohort studies have shown infected individuals develop an antibody response against SARS-CoV-2 that is maintained for 3–11 months [3–8]. Studies on endemic coronaviruses indicate immunity to these viruses wanes over time and individuals can become re-infected with the same strain [9–13]; however, it is unknown if SARS-CoV-2 immunity will similarly decline and/or if infected hosts will become susceptible to re-infection.

There have been several reports indicating immunity induced by infection is protective against SARS-CoV-2 re-infection in humans. One study found previously infected individuals had an 84% reduction in the risk of re-infection [14], while a second study found the risk was lowered by 95% [15]. Importantly, there have also been several reports of re-infection or breakthrough infection with SARS-CoV-2 [16–19]. In a subset of these cases, sequence analysis revealed the viral variant causing the secondary infection differed from the primary infection [16,17]. Given the rapid emergence of novel SARS-CoV-2 variants and variability in the exposure to the virus and the host response, it is difficult to accurately assess the durability of immunity to SARS-CoV-2 in humans. However, as SARS-CoV-2 becomes endemic, understanding the duration of immunity when viral evolution slows will be required to develop and guide vaccination strategies and public health measures.

While human studies are informative, animal studies can provide valuable insight into immune durability. In animal models, variables such as inoculation dose, viral strain, and timing of the secondary exposure can be controlled. In addition, susceptibility to re-infection can be directly assessed by challenging previously infected animals. Therefore, we used the Syrian hamster model of SARS-CoV-2 [20–22] to perform long-term re-infection studies. Hamsters were mock or SARS-CoV-2 infected and then given a secondary SARS-CoV-2 infection at 1-, 2-, 4-, or 6-months post-infection. After the primary SARS-CoV-2 infection, hamsters developed a robust antibody response that was maintained for at least 6 months (i.e. 168 days). Upon secondary SARS-CoV-2 infection, mock primary infected animals developed clinical illness and had high levels of replicating virus in the respiratory tract. All primary SARS-CoV-2 infected animals were protected from weight loss and re-infection, except one animal which had replicating virus in the nose and lungs after viral challenge at 4 months post-primary infection. Examination of the antibody response in this animal revealed it had comparable antibody titres to all other previously SARS-CoV-2 infected animals at the same time point. These findings demonstrate primary immunity and protection against SARS-CoV-2 is maintained for at least 6 months; however, even in the presence of high levels of antibodies, breakthrough infections can occur.

## Materials and methods

### Viruses and cells

SARS-CoV-2/USA/WA1/2020 was used for this study (BEI Resources, NR-52281, Lot 70036318). This virus was obtained at passage 4 and was passaged once on Vero E6 cells (ATCC). Viral titre was determined via tissue culture infectious dose 50% (TCID50) on Vero E6 cells. The virus stock was sequenced on an Illumina MiSeq platform and reads were analysed as previously described [23].

### Biocontainment and animal care and use

Experiments using SARS-CoV-2 were conducted in an animal biosafety level 3 enhanced (BSL3+) laboratory. This facility is approved for BSL3 + respiratory pathogen studies by the US Department of Agriculture and Centers for Disease Control. All animal studies were conducted in compliance with the Animal Care and Use Committee under protocol number 202001440.

### Study design

Equal numbers of male and female, 8-week-old, Syrian hamsters (Envigo, Haslett, MI) were divided into two groups (*n* = 70/group): (1) mock primary infection and (2) SARS-CoV-2 primary infection. Two days prior to the mock or SARS-CoV-2 infection, hamsters were sedated with ketamine (200 mg/kg) and xylazine (10 mg/kg) via intraperitoneal injection. Blood samples were collected via the gingival vein, and a subcutaneous transponder was inserted into the scruff (Bio Medic Data Systems). Hamsters were given a subcutaneous injection of atipamezole (1 mg/kg) and monitored until recovered. Blood samples were processed to recover serum and stored at −80°C.

For the mock or SARS-CoV-2 infection, hamsters (*n* = 70/group) were sedated with ketamine and xylazine and intranasally inoculated with 100uL of culture media or 10^5^ TCID_50_ SARS-CoV-2, respectively. After infection, clinical signs, body weight, and temperature were monitored for 14 days. On days 3 and 6 post primary infection, a subset of hamsters (*n* = 4/group/time point) were euthanized for collection of the nasal turbinate and lung tissues. One lung lobe was fixed in 10% formalin. The remaining lung and the nasal turbinates were stored at −80°C.

At 1, 2, 4, and 6 months post mock or primary SARS-CoV-2 infection, 14 animals from each original infection group were infected with 10^5^ TCID_50_ SARS-CoV-2. Serum was collected from a subset of hamsters (*n* = 10 mock or infected) bi-weekly for 168 days, and serum samples were collected from all animals one day prior to secondary infection. After virus inoculation, tissues were collected from a subset of animals at 3- and 6-days post-infection (dpi) (*n* = 4/group/time point). Weight loss was monitored daily (*n* = 6/group) through 14 dpi and animals were euthanized via CO_2_ asphyxiation.

### Viral titrations

Lung and turbinate samples were weighed and homogenized in 2% FBS-DMEM using the Omni Tissue Homogenizer and centrifuged at 1000×*g* for 10 min at 4°C. Serial 10-fold dilutions of homogenate supernatants were added to 96-well plates of Vero E6 cells. Plates were incubated at 37°C and scored for cytopathic effect at 96 h post-infection. TCID50 was calculated using the method of Reed and Muench [24].

### Assessment of the antibody response

To quantify anti-SARS-CoV-2 antibodies, indirect ELISA was performed as previously described [25] with the following modifications: ELISA plates (Nunc) were coated with 2.0 μg/mL RBD, S protein, or N protein (Sino Biological) in sodium bicarbonate buffer. Plates were read on a SpectraMax iD3 (Molecular Devices) plate reader. Absorbance readings three standard deviations above the mean day 0 value were considered positive, and titres were reported as the highest serum dilution above this cut-off value. To quantify neutralizing antibodies, microneutralization assays were performed using hamster serum as previously described [23].

### Histopathology

Cross sections of each formalin fixed left lung lobe were placed in cassettes and processed using the Tissue-Tek® VIP^TM^ 5 Vacuum Infiltration Processor (Sakura Finetek). Tissues were embedded in paraffin using the Tissue-Tek® TEC^TM^ 5 Embedding Console (Sakura Finetek). Sections (5 μm) were cut from the paraffin blocks and placed on slides. Slides were stained with haematoxylin and eosin using the Leica Autostainer XL (Leica Biosystems Inc) and scored by a board-certified veterinary pathologist using established methods [26,27].

### Multiplex immunoassay

IgG antibody binding titres were determined by an electrochemiluminescent-based multiplex immunoassay (Meso Scale Discovery). SARS-CoV-2 panels 11, 13, 22 and 23 were used to detect binding antibodies against the RBD and S protein for the Wuhan strain and multiple variants of concern. These variants included B.1.1529, B.1.617.2, B.1.617.1, P.1, B.1.351 and B.1.1.7. Plates were pre-coated with RBD or spike antigens, and hamster serum samples were diluted at 1:100,000 and added to the plates. These kits are specific to human antibodies; thus, the following modifications were performed. Biotin conjugated goat anti-hamster IgG antibody (ThermoFisher) was added in 1:20,000 dilutions. To detect the RBD and S protein hamster antibodies, SULFO-TAG^TM^ streptavidin antibody (Meso Scale Discovery) and SULFO-TAG^TM^ Donkey Anti-Goat antibody (Meso Scale Discovery) at a 1:500 dilution were added to the plates, respectively. MSD Gold Read Buffer B was added prior to reading on MSD plate reader. Data was analysed using Discovery Workbench.

### Statistical analysis

Body weights were compared between groups using a 2-way ANOVA with Sidak’s multiple comparison test. Non-parametric pairwise analysis for RBD and S protein antibody titres were performed by Wilcoxon matched-pairs signed rank test. Antibody half-life calculations were performed using a one phase exponential decay nonlinear regression model. Histopathological tissue scores from mock and infected animals on 3 or 6 dpi were compared by Mann–Whitney test. Analyses were performed using GraphPad Prism (v 9.2.0) with a *p*-values < 0.05 considered significant.

## Results

### Primary SARS-CoV-2 infection of Syrian hamsters results in viral replication in the upper and lower respiratory tract, weight loss, and lung pathology

**Figure 1.**
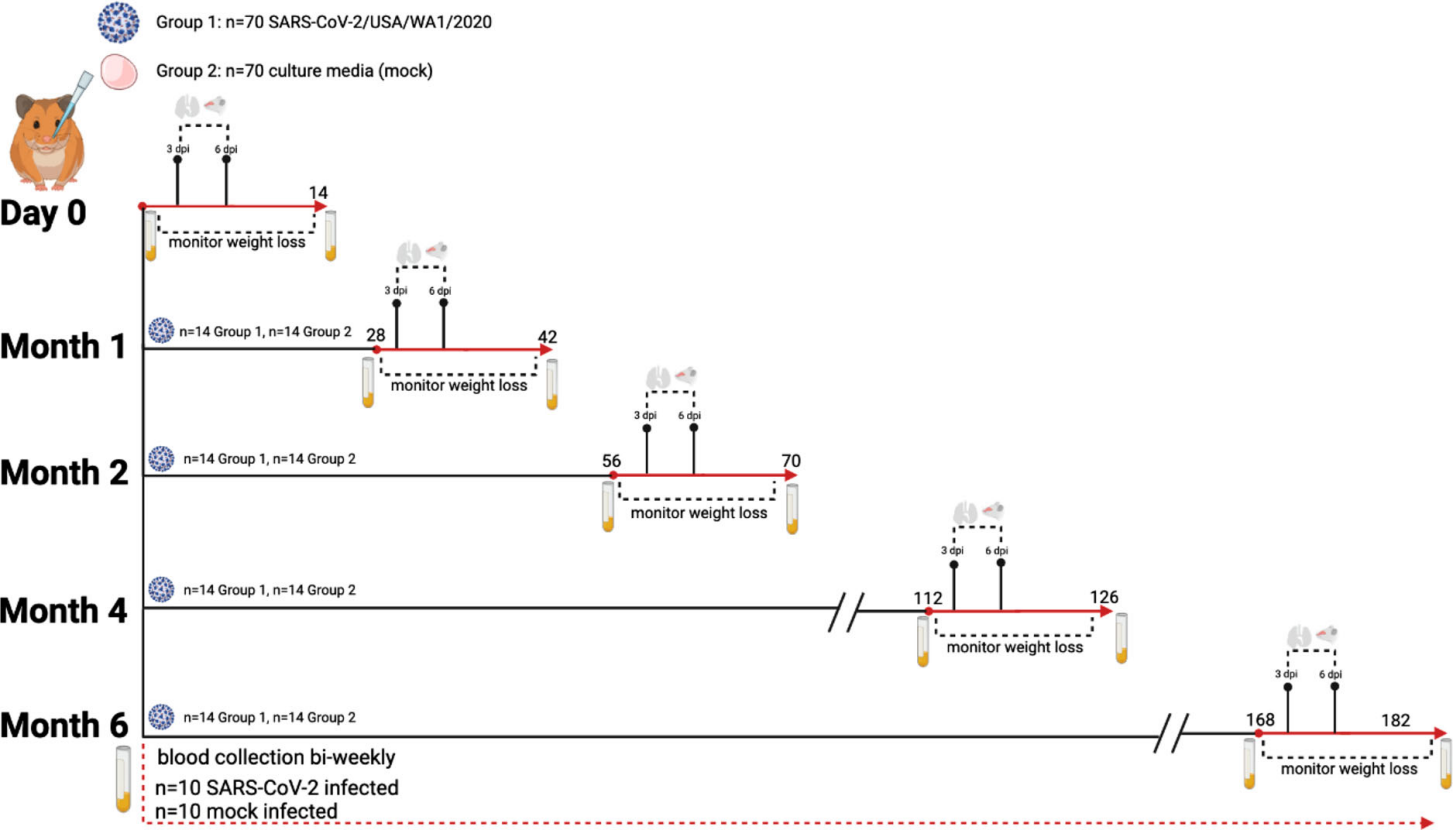
Schematic of experimental design*.* Hamsters (*n* = 70/group) were given a primary mock or SARS-CoV-2 infection (10^5^ TCID50 USA/WA1/2020). At 3 and 6 dpi, lung and nasal turbinates were collected (*n* = 4/group/time point), and weight loss was monitored in the remaining animals for 14 days. At the time of secondary challenge (1, 2, 4, or 6 months post-primary infection), 14 hamsters from each original group (SARS-CoV-2 infected or mock) were intranasally challenged with SARS-CoV-2 (10^5^ TCID50 USA/WA1/2020). Serum samples were collected from a subset of hamsters (*n* = 10 infected and mock) for the duration of the study until day 168.

**Figure 2.**
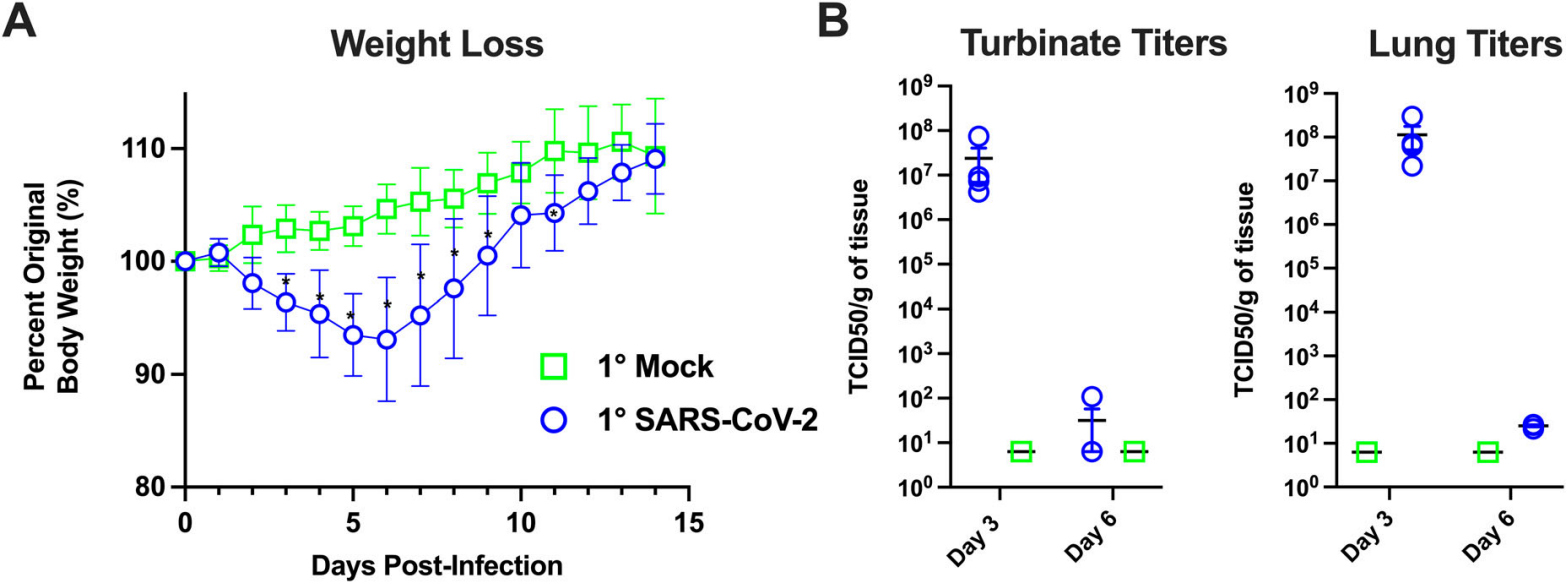
Primary SARS-CoV-2 infection causes weight loss and viral replication in the respiratory tract. (A) Body weight changes in hamsters after primary mock (green, *n* = 6) or SARS-CoV-2 infection (blue, *n* = 6). * denotes *p*≤0.01 between groups. (B) Viral titres in nasal turbinates and lungs on day 3 and 6 post-primary mock (green squares) or SARS-CoV-2 infection (blue circles) (*n* = 4/group/time point).

**Figure 3.**
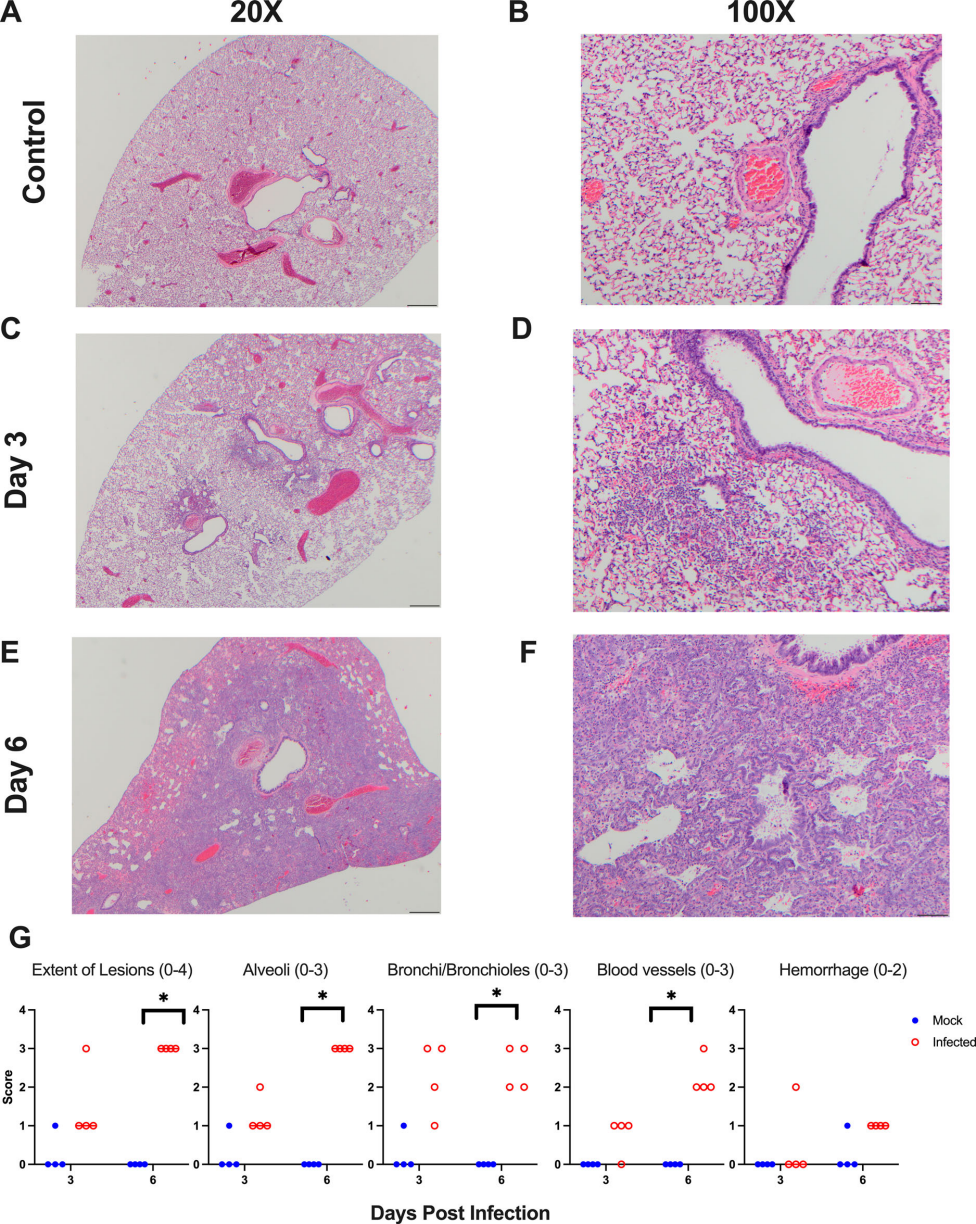
Primary SARS-CoV-2 infection causes lung pathology consistent with COVID-19*.* Shown are H & E stained lung sections imaged at 20× and 100× magnification, scale bars are 500 and 100 μm respectively. (A,B) Lungs taken from an uninfected control animal; no lesions observed in day 3 or 6 animals, only day 3 animals are displayed. (C,D) Lung, 3 days post-primary SARS-CoV-2 infection: Prominent pathological features include a mild mononuclear cell infiltrate centred around bronchioles, minimal perivascular infiltrate, bronchiolar epithelial degeneration, and alveolar oedema. (E,F) Lung, 6 days post-SARS-CoV-2 infection: Prominent features include marked type II pneumocyte hyperplasia, robust bronchiolar and interstitial mononuclear cell infiltrate, perivascular oedema, and moderate alveolar oedema. (G) Lungs were scored on the extent of lesions (0–4), alveoli (0–3), bronchi/bronchioles (0–3), blood vessels (0–3), and haemorrhage (0–2). * denotes *p *≤ 0.05 between scores for each group.

At the onset of this study, we mock-infected (*n* = 70) hamsters with culture media, and we infected hamsters (*n* = 70) with 10^5^ TCID50 of SARS-CoV-2 (Figure 1). The mock-infected animals gained weight while the SARS-CoV-2 infected animals exhibited weight loss which peaked at ∼7–10% of body weight at 6 dpi (Figure 2(A)). We next evaluated the extent of viral replication in the upper and lower respiratory tract. As shown in Figure 2(B), at 3 dpi viral titres in the nasal turbinates and lungs ranged from 10^6^–10^8^ TCID50/g, respectively. By day 6, viral titres were reduced to low levels indicative of viral clearance. Histopathological analysis of lung sections at 3 dpi revealed a mild mononuclear cell infiltrate around the bronchioles with subtle perivascular infiltrate, bronchiolar epithelial damage, and alveolar oedema (Figure 3(C,D)). By 6 dpi, lung pathology consisted of severe type II pneumocyte hyperplasia with alveolar and perivascular edema and a robust bronchiolar and interstitial mononuclear cell infiltrate (Figure 3(E,F)). Mock-infected hamsters exhibited no pathological abnormalities (Figure 3(A,B)). SARS-CoV-2 infected hamsters had significantly higher pathology scores than mock-infected hamsters on 6 dpi for extent of lesions, alveoli, bronchi, and blood vessels (Figure 3(G)). Collectively, our findings are consistent with published reports [20–22,28] demonstrating SARS-CoV-2 infected hamsters experience a productive infection and COVID-19 like disease.

### Antibodies against SARS-CoV-2 were maintained for 6 months post-infection

**Figure 4.**
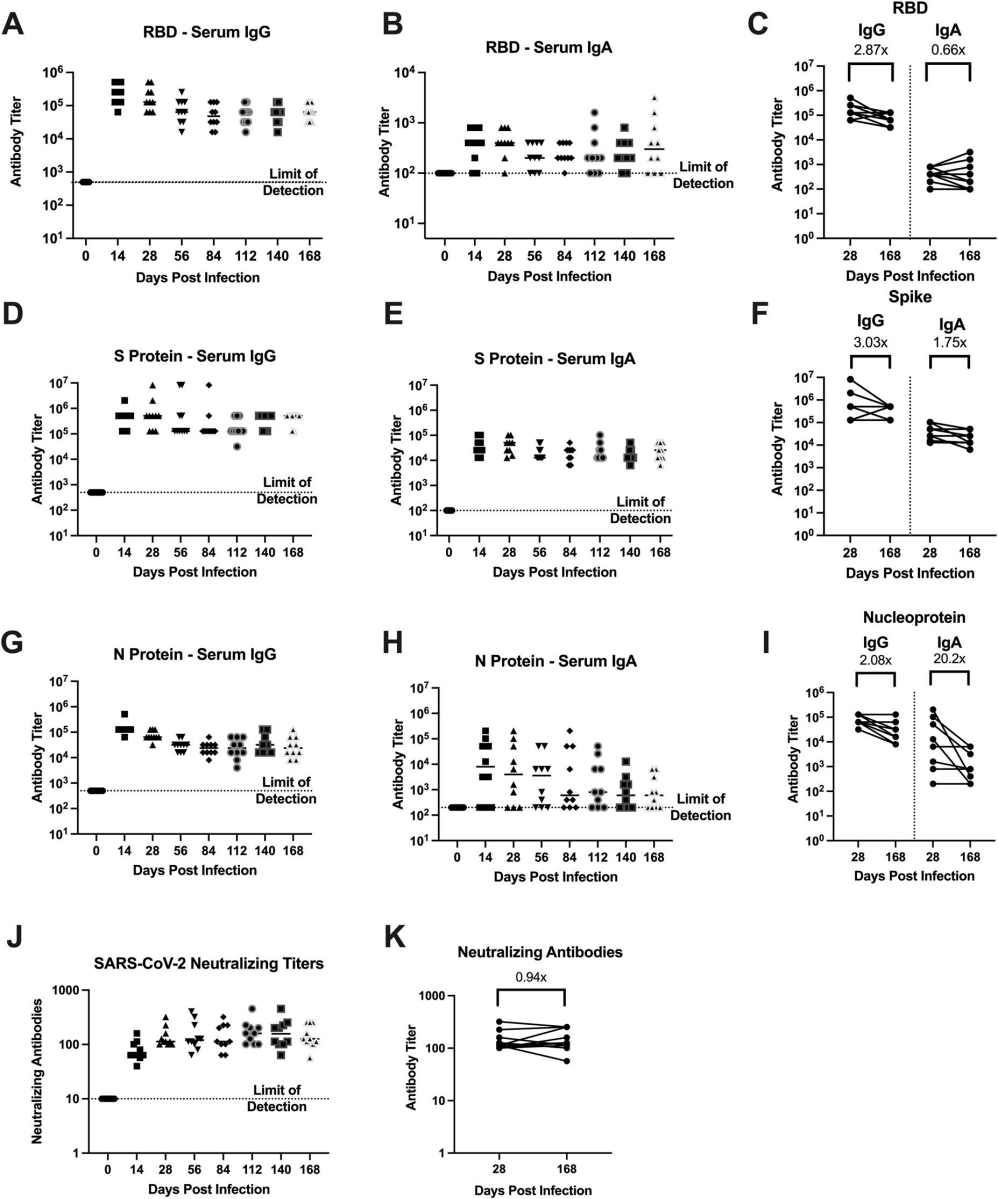
SARS-CoV-2 infected hamsters maintain antibodies against SARS-CoV-2 for 6 months post-infection. Assessment of the binding antibody response from day 0 to 168 in SARS-CoV-2 infected hamsters by ELISA (*n* = 10/time point). Serum levels of (A) IgG and (B) IgA binding antibodies, and (C) fold change of serum levels of IgG and IgA antibodies against the RBD from SARS-CoV-2 infected hamsters. Serum levels of (D) IgG and (E) IgA antibodies, and (F) fold change of serum levels of IgG and IgA antibodies against the S protein from infected hamsters. Serum levels of (G) IgG and (H) IgA antibodies, and (I) fold change of serum levels of IgG and IgA antibodies against the N protein from infected hamsters. (J,K) Neutralizing antibody titres in serum collected from SARS-CoV-2 infected animals between days 0 and 168 (*n* = 10).

To determine the longevity of the antibody response, using serum samples collected over 168 days (i.e. 6 months), we evaluated the IgG and IgA antibody response by ELISA against the RBD, S protein, and N protein. No SARS-CoV-2 antibodies were detected in the hamsters prior to infection (day 0, Figure 4) or in any of the mock-infected hamsters prior to secondary SARS-CoV-2 challenge (data not shown). After the primary SARS-CoV-2 infection, anti-RBD IgG titres peaked at 14 and 28 dpi (Figure 4(A)). On day 56 antibody titres declined but were maintained for the remainder of the study (Figure 4(A)). Between 28 and 168 dpi antibody titres against the RBD decreased by 2.87-fold (Figure 4(C)). IgG titres against the S protein followed the same pattern with peak titres at 14 and 28 dpi, followed by a decline at 56 dpi. Antibody titres against the S protein decreased by 3.03-fold over the course of the study (Figure 4(D,F)). IgG titres against the N protein peaked at 14 dpi and then declined, but were maintained through the length of the study, decreasing by 2.08-fold between 28 and 168 dpi (Figure 4(G,I)). Using a one phase exponential decay model, we estimated the half-life of the IgG responses to be 134, 380, and 145 days against the RBD, S protein, and N protein, respectively.

The IgA antibody response mirrored the IgG response although titres were lower and more variable. IgA titres against the RBD were detected by 14 dpi, however, not all animals developed a response. As a result, a 0.66-fold decrease in IgA titres against the RBD was observed between 28 and 168 dpi (Figure 4(B,C)). In contrast, all infected hamsters developed an IgA response against the S protein by 14 dpi and titres were maintained at 28 dpi but slowly declined by 1.75-fold by the end of the study (Figure 4(E,F)). Last, we evaluated the IgA response against the N protein. Anti-N IgA antibody titres were highly variable, although we observed a 20.2-fold decrease between 28 and 168 dpi (Figure 4(H,I)). As several animals did not develop an IgA antibody response against the RBD or N protein, and there was minimal waning of the IgA response against the S protein, we could not estimate the half-life for IgA antibodies.

We also examined the neutralizing ability of antibodies in the serum. All SARS-CoV-2 infected hamsters developed neutralizing antibodies that were maintained for the duration of the study, with only a 0.94-fold reduction in neutralizing titres from 28 to 168 dpi (Figure 4(J,K)). As observed for the IgA antibody response, due to minimal waning, we could not estimate the half-life. Overall, infected hamsters mounted a robust anti-RBD, anti-S, and anti-NP IgG response, a stable IgA response towards the full-length S protein with a variable response directed towards the RBD and N protein. A proportion of these antibodies exhibited neutralizing activity and were maintained through the entirety of the study.

### Previously infected hamsters were protected from disease upon re-infection, although breakthrough infection was observed at 4 months post-primary infection

**Figure 5.**
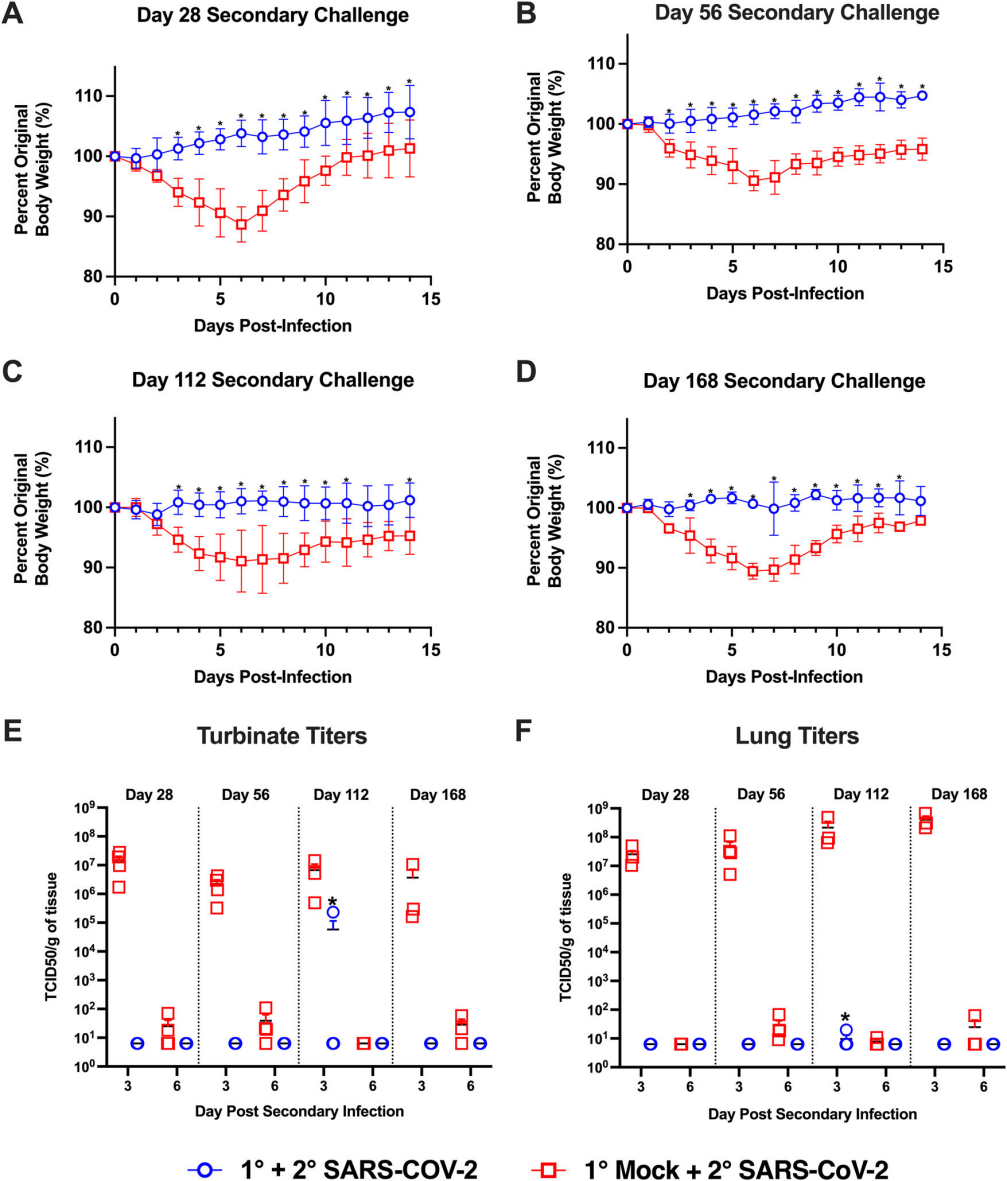
Hamsters are protected from weight loss upon re-challenge with SARS-CoV-2. Body weight changes in hamsters after challenge at designated time points (panels A–D) after the primary infection in previously mock-infected hamsters challenged with SARS-CoV-2 (red, *n* = 6) compared to previously SARS-CoV-2 infected animals given a secondary SARS-CoV-2 infection (blue, *n* = 6). * denotes *p *≤ 0.01 between groups. Panels (E,F) show viral titres in nasal turbinate and lung samples in animals (*n* = 4/group/time point) given a primary mock infection followed by a SARS-CoV-2 infection (red squares) and from animals given a primary SARS-CoV-2 infection and then re-challenged with SARS-CoV-2 (blue circles). Breakthrough infection was observed in one hamster denoted by * following the 4-month secondary infection.

Next, we sought to evaluate if immunity conferred by prior infection would protect against re-infection, and if this protection would be maintained over several months. Prior studies demonstrated hamsters are susceptible to re-infection at 1-month post-primary infection when challenged with a variant strain (i.e. heterologous challenge) [29,30]. To evaluate the potential for re-infection with a homologous isolate, we re-challenged cohorts of previously infected and mock-infected animals with the USA/WA1/2020 isolate at 1, 2, 4, and 6 months post-primary infection (Figure 1). As shown in Figure 5(A–D), when previously mock-infected animals were given a secondary SARS-CoV-2 infection, regardless of the interval between the mock and secondary infection, all hamsters exhibited weight loss that peaked at day 6 with a loss of approximately 10% body weight. In contrast, upon secondary SARS-CoV-2 challenge of previously infected animals, none of the animals lost weight at any time point. This demonstrates prior infection with SARS-CoV-2 confers long-term protective immunity against clinical illness upon repeat exposure to the same isolate.

Upon analysis of the viral titres in the nasal turbinates and lung following secondary SARS-CoV-2 challenge, the viral titres at 3 and 6 dpi in the primary mock-infected hamsters were comparable to those seen in SARS-CoV-2 infected animals (compare Figure 2(B,C) to Figure 5(E,F)). Replicating virus in the nasal turbinates and lungs was not detected following secondary SARS-CoV-2 challenge for any of the primary SARS-CoV-2 infected hamsters at 1, 2, and 6 months post-primary infection (Figure 5(E,F)). However, one hamster in the 4-month secondary challenge group exhibited viral replication in the nasal turbinates (2.32 × 10^5^ TCID50/g) and lungs (1.98 × 10^1^ TCID50/g) at 3 dpi (Figure 5(F)). As the virus had to be maintained in this animal for at least 3 days, this indicates the animal experienced a productive breakthrough infection with viral replication throughout the respiratory tract. Thus, while all animals were protected from weight loss, immunity may be incomplete and breakthrough infections may again become possible at 4-months post-primary infection.

**Figure 6.**
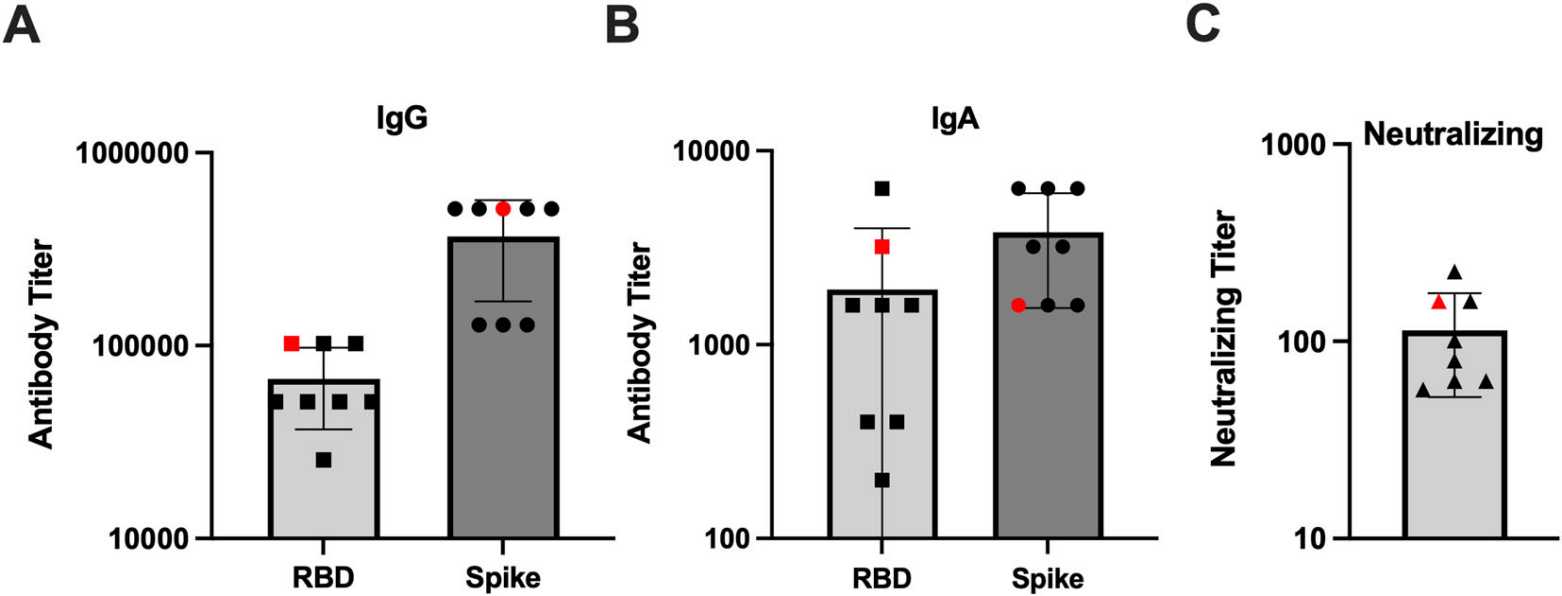
IgG and IgA binding and neutralizing antibody titres in the 4-month secondary challenge group*.* Prior to secondary virus challenge, blood samples were collected from all animals to evaluate the antibody response in the event of a breakthrough infection. Shown are antibody titres in previously SARS-CoV-2 infected animals given a secondary SARS-CoV-2 infection at 4 months post-primary infection. One animal denoted by the red symbol developed a breakthrough infection. Antibody titres against the RBD and S proteins by (A) IgG and (B) IgA ELISA, and (C) neutralizing titres determined via microneutralization assay.

Importantly, we collected serum from all the hamsters prior to the secondary challenge. Using this serum, we compared the antibody response in the animal that experienced a breakthrough infection to the other primary infected animals in the 4-month challenge cohort. As shown in Figure 6, the hamster that experienced a breakthrough infection had IgG and IgA antibody titres against the RBD and S protein that were equivalent or greater than the other hamsters challenged at 4 months post-primary infection. These antibodies were also capable of neutralizing infectious SARS-CoV-2 (Figure 6(C)). This indicates that breakthrough infection occurred even in the presence of a robust antibody response.

### Prior infection induced a durable and cross-reactive antibody response against SARS-CoV-2 variants of concern

**Figure 7.**
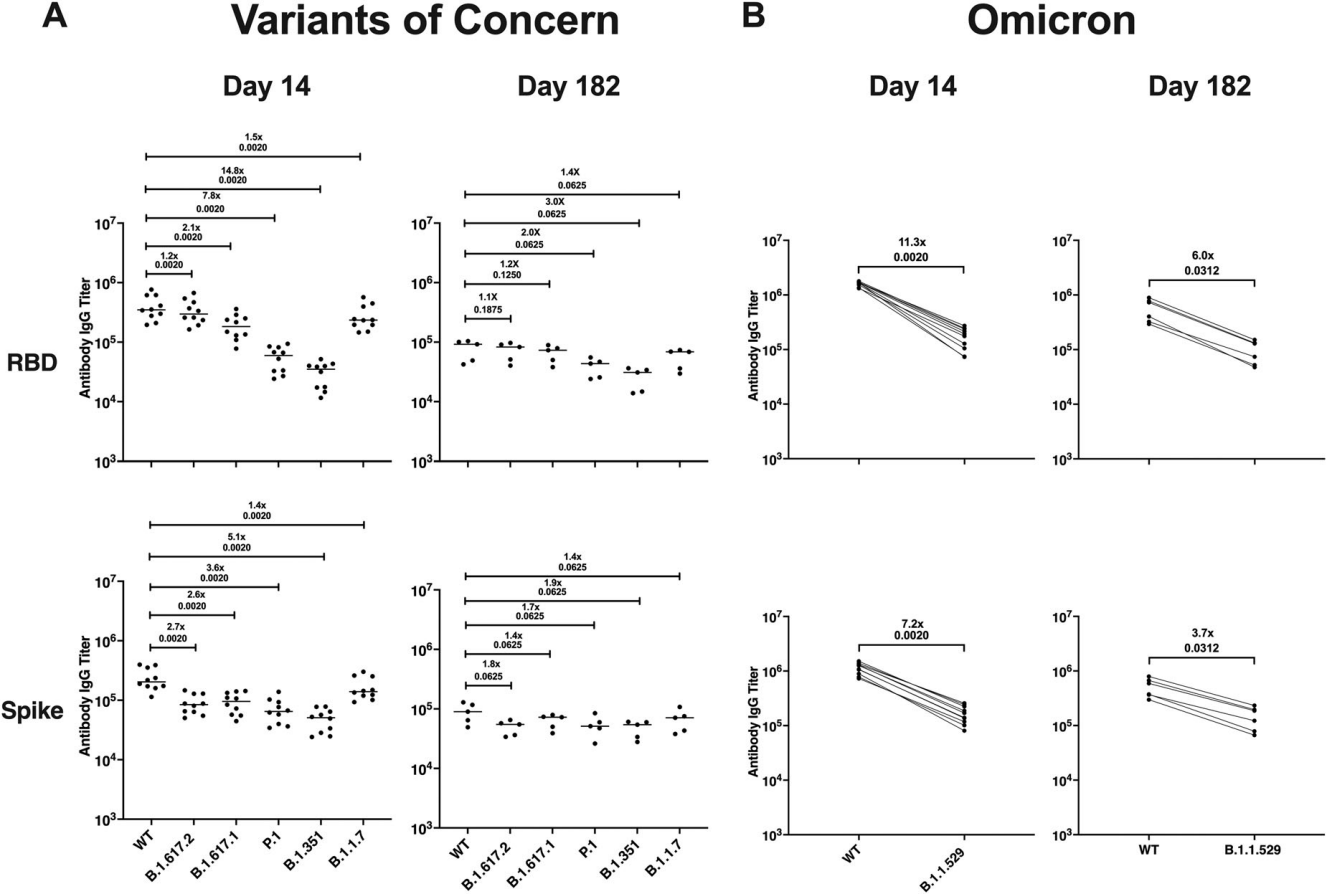
Infection with SARS-CoV-2/USA/WA1 induces a durable and cross-reactive antibody response against variants of concern. Serum samples collected on 14 (*n* = 10) and 182 dpi (*n* = 6) from an additional group of hamsters given a primary SARS-CoV-2 infection were used in a multiplex immunoassay. (A) Antibody binding and fold change against the RBD and S protein for both the Wuhan-1 (WT) and VOCs including B.1.617.2, B.1.617.1, P.1, B.1.351, and B.1.1.7. (B) Antibody binding and fold change against the RBD and S protein for Wuhan-1 (WT) and B.1.1.529. Non-parametric pairwise analysis for RBD and S specific IgG titres were performed by Wilcoxon matched-pairs signed-rank test. *p*-values are displayed below the fold-change value.

To determine if the antibodies induced against the USA/WA1/2020 isolate would cross-react to numerous variants of concern (VOCs), we performed a multiplex immunoassay using serum from an additional cohort of SARS-CoV-2 infected hamsters that were maintained out to 182 dpi. We compared antibody binding of serum collected at 14 and 182 dpi using SARS-CoV-2 panels for both the RBD and S protein comparing the Wuhan-1 strain, which is closely related to the WA1/2020 isolate, to VOCs including B.1.617.2 (delta), B.1.617.1 (kappa), P.1 (gamma), B.1.351 (beta), B.1.1.7 (alpha), and B.1.1.529 (omicron). For delta, alpha, kappa, gamma, and beta, antibody binding to the RBD was 1.2–14.8–fold lower than to the Wuhan-1 strain on day 14, and on day 182, there was a 1.1–3.0-fold reduction in RBD-binding antibodies (Figure 7(A)). Antibody binding to the S protein was 1.4–5.1-fold lower for alpha, kappa, delta, gamma, and beta than that of Wuhan-1 on day 14, and this difference was reduced to 1.4–1.9-fold on day 182 (Figure 7(A)). Importantly, antibody binding to the RBD of the omicron variant was 11.3-fold lower than to Wuhan-1 on day 14 and 6.0-fold lower on day 182 (Figure 7(B)). Antibody binding to the omicron S protein was 7.2-fold lower than that of Wuhan-1 on day 14, and this difference was reduced to a 3.7-fold on day 182 (Figure 7(B)). These findings suggest a primary infection with the WA1/2020 strain confers a durable antibody response against VOCs. However, while antibody cross-reactivity is suggestive of protection, the breakthrough infection we observed at 4 months post-primary infection indicates antibody-mediated immunity may not confer complete protection and the use of antibody titre alone to infer protection may not be accurate.

## Discussion

Over the course of the pandemic, there have been reports of re-infection in individuals confirmed to have a prior SARS-CoV-2 infection [16–19]. These findings suggest the previous infection may not confer complete protection and it is unclear if re-infection is due to natural immunity waning over time or the emergence of viral variants that escape pre-existing immunity. Developing an understanding of the durability of immunity is required to guide public health decisions and mitigate the spread of future epidemic waves of SARS-CoV-2. Therefore, we assessed the longevity of immune protection against re-infection using the Syrian hamster model of SARS-CoV-2. Animals were given a primary mock or SARS-CoV-2 infection and then received a secondary SARS-CoV-2 infection at 1–6-month intervals post-primary infection. We found all previously SARS-CoV-2 infected hamsters were protected against weight loss upon re-infection; however, we detected a breakthrough infection in one animal challenged at 4 months post-primary SARS-CoV-2 infection. To induce natural immunity, we infected a large cohort of hamsters with SARS-CoV-2 and these animals exhibited significant body weight loss associated with viral replication in the upper and lower respiratory tract (Figure 2(A,B)). Histopathological hallmarks of viral pneumonia were consistent with COVID-19 in humans and were observed at 6 dpi (Figure 3). After resolving the acute SARS-CoV-2 infection, hamsters developed a robust antibody response with high titres of serum IgG and variable IgA antibody titres, which collectively exhibited neutralizing activity (Figure 4). These findings are consistent with reports by other groups on primary SARS-CoV-2 infection in hamsters [20–22,28]; however, we extend these findings by demonstrating antibody titres are maintained for at least 6 months.

IgG antibodies against the RBD, S and N proteins had a half-life of 134, 380, and 145 days, respectively. Importantly, the estimate of the anti-RBD IgG antibody half-life is comparable to that reported in humans of 116 days [31]; however, our estimate for the half-life of antibodies against the S protein was substantially longer than 126 days reported in humans. This discrepancy may be due to greater heterogeneity in the initial virus dose and/or greater genetic diversity in humans resulting in a more varied data set and shorter estimated half-life.

The role of immunity induced by prior infection against re-infection with variant strains of SARS-CoV-2 has been evaluated in hamsters [29,30]. In these studies hamsters were protected against weight loss when challenged 21 or 28 days post-primary infection with a heterologous SARS-CoV-2 variant; however, between days 1–5 post-secondary challenge, replicating virus could be recovered from a proportion of previously infected animals [29,30]. While using different viruses to prime and re-infect hamsters allowed for the assessment of protection against emerging strains, using a heterologous challenge limits assessment of immune longevity. Thus, by utilizing a homologous virus challenge at 1–6-month intervals, we directly assessed the durability of immunity. Consistent with a previous report showing hamsters were protected from clinical illness when re-infected at 1 month following initial infection [29], our previously SARS-CoV-2 infected hamsters were protected from weight loss upon secondary challenge at all time points (Figure 5(A–D)). Except for a single animal, all the previously SARS-CoV-2 infected hamsters were protected from re-infection as replicating virus could not be recovered from the lungs and nasal turbinates. In one hamster challenged at 4-months post-primary infection, moderate and low titres of replicating virus were recovered from the nasal turbinate and lungs, respectively (Figure 5(E,F)). When antibody titres were examined in this animal prior to secondary infection, there were high titres of IgG and IgA antibodies against both the RBD and S protein, and these antibodies displayed neutralizing activity (Figure 6). Comparison of Figure 4 to Figure 6 also indicates the antibody titres were comparable to those observed 4 months post-primary infection in the animals monitored for 6 months. These findings are consistent with a recent study that evaluated re-infection of hamsters with the WA1 strain at 4 months post-infection. In this study, virus could not be recovered in the nasal wash, but one animal had replicating virus in the lungs at 5 dpi despite high levels of anti-RBD IgG antibodies [32]. Collectively, our findings indicate that while the antibody response to SARS-CoV-2 in hamsters may be long-lasting and protective against clinical illness, immunity may be incomplete, and animals may again become susceptible to infection. However, as only one breakthrough infection was observed, our findings demonstrate in most animals’, prior infection confers long-term immunity which is protective against disease and infection with a homologous SARS-CoV-2 strain.

We further demonstrated hamsters infected with the USA/WA1/2020 isolate produced antibodies that cross-react with multiple viral variants including the Omicron variant (Figure 7). While this may be suggestive of protection against clinical disease, our detection of a breakthrough infection indicates the use of antibody titres to determine when a host may again become susceptible to SARS-CoV-2 may be imperfect. As hamster-specific reagents are developed, it will be critical to assess the longevity of the cellular immune response in parallel with the antibody response as discrepancies in these responses may indicate susceptibility to re-infection. While our results show re-infection is possible, our findings demonstrate hamsters have long-term immunity and protection against a homologous challenge and immunity does not wane substantially over 6-months.

Of note, the viral stock used in our studies was obtained at passage 4 and passaged once on Vero E6 cells. As multiple reports have demonstrated repetitive passaging of SARS-CoV-2 in Vero E6 cells can lead to mutations in the furin cleavage site (FCS) [33,34], we performed next-generation sequencing of the stock. The SARS-CoV-2 stock had mutations in the FCS of 93% of the virus population (Supplemental Table 1). However, infection of hamsters with this stock caused disease indicated by weight loss and histopathological findings. As hamsters were given a dose of 10^5^ TCID50, these animals received greater than or equal to 10^3^ TCID50 of virions with an intact FCS. Hamster infectious dose studies with the USA/WA1/2020 isolate have shown a dose of 10^3^ TCID50 is sufficient to induce 5–10% weight loss, while a dose of 10^2^ TCID50 induces minimal (i.e. 1–3%) weight loss [24]. As primary SARS-CoV-2 infection in our hamsters resulted in ∼7–10% weight loss, this suggests the hamsters received a dose of at least 10^3^ TCID50 of virions with an intact cleavage site.

Collectively our studies demonstrate hamsters are protected from clinical disease upon re-challenge with a homologous virus for at least 6 months. After primary infection, animals develop stable titres of IgG and neutralizing antibodies; however, we detected one breakthrough infection in an animal with high antibody titres. Thus, while prior infection protects against disease, it may not provide complete protection or sterilizing immunity. Given hamsters mimic the clinical presentation of COVID-19, human patients that develop a moderate to severe disease may also be protected from illness following a second exposure to SARS-CoV-2 for up to 4–6 months. Our findings indicate immunity to SARS-CoV-2 and protection against re-infection does not decline rapidly over time and hosts are protected from severe disease upon repeat exposure to homologous SARS-CoV-2 strain.

## Supplementary Material

Supplemental Material

## Dedication

We would like to dedicate this manuscript to Talia Heinly whose enthusiasm for research and life will always be remembered.
