# Supplementary material for: Immune durability and protection against SARS-CoV-2 re-infection in Syrian hamsters
Source: Emerg Microbes Infect. 2022 Apr 18;11(1):1103–14. doi: 10.1080/22221751.2022.2058419 (PMC9037228; doi:10.1080/22221751.2022.2058419)
Supplement: Supplemental Material [file TEMI_A_2058419_SM8724.docx]

**Supplementary Materials**

***Supplemental Table 1. Quasi species present in SARS-CoV-2/USA/WA1/2020 viral stock***

| **Genomic position** | **Original Nucleotide** | **Polymorphic Nucleotide** | **Polymorphism Frequency** | **Gene** | **Amino Acid Polymorphism** | **Notes** |
| --- | --- | --- | --- | --- | --- | --- |
| 17823 | C | A | 20.42% | orf1ab | Q5855K |  |
| 22202 | A | G | 25.00% | S | D215G |  |
| 22292 | A | G | 38.47% | S | H245R |  |
| 22478 | C | T | 21.06% | S | T307I |  |
| 23521 | C | T | 13.29% | S | H655Y |  |
| 23580 | T | 15nt deletion  (-CAGACTCAGACTAAT) | 1.68% | S | 5 amino acid deletion  (-QTQTN) | Adjacent to FCS |
| 23589 | G | 36nt deletion  (-ACTAATTCTCCTCGGCGGGCACGTAGTGTAGCTAGT) | 35.33% | S | 36 amino acid deletion  (-TNSPRRARSVAS) | FCS |
| 23592 | T | 30nt deletion  (-AATTCTCCTCGGCGGGCACGTAGTGTAGCT) | 0.56% | S | 10 amino acid deletion  (-NSPRRARSVA) | FCS |
| 23592 | T | 21nt deletion  (-AATTCTCCTCGGCGGGCACGT) | 1.12% | S | 7 amino acid deletion  (*/-NSPRRAR) | FCS |
| 23602 | C | T | 19.19% | S | R682W | FCS |
| 23603 | G | T/A | 30.18% | S | R682L/R682Q | FCS |
| 23614 | A | G | 6.76% | S | S686G | FCS |
| 23622 | T | 9nt deletion  (-AGTCAATCC) | 0.19% | S | 3 amino acid deletion  (-SQS) | Adjacent to FCS |
|  | | **Total Furin deleted/mutated viruses:** | **93.14%** |  | | |

Notes: FCS denotes furin cleavage site
